# Sustainable packaging materials for fermented probiotic dairy or non-dairy food and beverage products: challenges and innovations

**DOI:** 10.3934/microbiol.2024017

**Published:** 2024-05-08

**Authors:** Dali Vilma Francis, Divakar Dahiya, Trupti Gokhale, Poonam Singh Nigam

**Affiliations:** 1 Department of Biotechnology, Birla Institute of Technology and Science, Pilani, Dubai Campus, Dubai International Academic City, PO Box 345055 UAE; 2a Wexham Park Hospital, Wexham Street, Slough SL2 4HL, UK; 2b Current address: Haematology and Blood Transfusion, Basingstoke & North Hampshire Hospital, Basingstoke RG24 9NA, UK; 3 Biomedical Sciences Research Institute, Ulster University, Coleraine BT52 1SA, UK

**Keywords:** fermented, dairy, products, packaging, sustainability, technologies, probiotic, food, beverages, environment

## Abstract

The food and beverage packaging industry has experienced remarkable growth in recent years. Particularly the requirement for appropriate packaging materials used for the sale of fermented products is boosted due to the rising acceptance of economical functional foods available to consumers on the shelves of their local supermarkets. The most popular nutraceutical foods with increased sales include natural yogurts, probiotic-rich milk, kefir, and other fermented food and beverage products. These items have mainly been produced from dairy-based or non-dairy raw materials to provide several product options for most consumers, including vegan and lactose-intolerant populations. Therefore, there is a need for an evaluation of the potential developments and prospects that characterize the growth of the food packaging industry in the global market. The article is based on a review of information from published research, encompassing current trends, emerging technologies, challenges, innovations, and sustainability initiatives for food industry packaging.

## 1. Introduction

In the elaborate intersection of tradition and innovation, where the research has focused on techniques of food fermentation uniting it with its innovative packaging technology, is the cooperative work of two popular industries to provide quality food in a good acceptable condition and to reach wider locations in a safer protected way. The amount of published research shows great interest in both aspects, captivating the world of fermented products and delivering practical and sustainable packaging material. Both industries, characterized by their dynamism and constant evolution, have claimed a prominent position in the highlight. Their progress can be attributed to the global surge in appreciation for functional nutraceutical food and beverage products including bio-yogurt, kefir, fermented milk, and a range of probiotic non-dairy equivalents [1]. These products, cherished for their potential health benefits, have transcended the realm of mere culinary choices to become dietary bases for vegan and non-vegan consumers across the globe [2].

There is an ever-increasing need for sustainable packaging materials for fermented probiotic dairy or non-dairy food and beverage products. The reason behind the search for cheaper and sustainable packaging material for products in the food and beverage industry is the shorter shelf-life of such products and the importance of their uncontaminated safer transportation to local supermarkets from industrial production units [3]–[6]. The global market for the sale of functional food has increased globally as these products are classified as nutraceuticals. A diverse range of dairy or non-dairy food items requires proper effective packaging for those products which have undergone natural or controlled fermentation processes, often facilitated by specific strains of beneficial microbial strains of probiotic bacteria and yeast [7]–[11]. These transformations in optimized fermentation systems produce a wide selection of products with desired distinctive flavors, textures, and nutritional attributes.

In an era marked by preferences of consumers recognizing the tastes of functional food and beverages, the packaging of fermented dairy products has assumed an indispensable role in shaping their market potential. Beyond its fundamental responsibility of preserving the freshness and integrity of these delicate dairy delicacies, packaging serves as an ambassador, imparting essential information to consumers. It conveys critical details about nutritional content, sourcing origins, and production methods. Moreover, in an age where environmental consciousness has grown, the call for sustainable packaging solutions has become exceptionally demanding.

The following sections describe a comprehensive review of potential developments and prospects of novel packaging materials for food products with sustainability possibilities. It offers a value awareness to food industry professionals, packaging designers, manufacturers, and all stakeholders. Information has been presented on current packaging trends, exploring the options of emerging technologies, and offering consumer-friendly sustainability initiatives.

## 2. Understanding the life cycle of fermented products packaging

**Figure 1.**
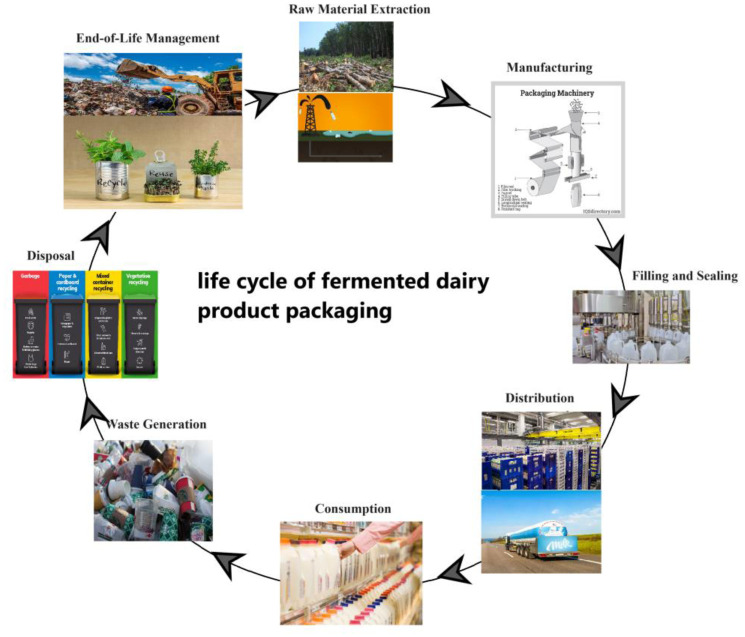
Life cycle of food products packaging: Environmental considerations.

The life cycle of fermented food product packaging encompasses several key phases, each with its unique environmental considerations. It begins with the extraction of raw materials and extends through manufacturing, distribution, consumer use, and ultimately, management of end-of-life materials including by-products and wastes. Sustainable practices, such as using recyclable or compostable materials, optimizing packaging design, and reducing energy use during manufacturing, are vital for minimizing the environmental impact and aligning with consumer preferences for eco-friendly options [12]. At each stage, factors like material sourcing, energy consumption, emissions, and waste generation impact the sustainability and environmental footprint of the packaging [13]. Understanding and addressing these factors throughout the life cycle are essential for developing packaging solutions that prioritize both product quality and environmental responsibility. Figure 1 presents an illustrative representation of the life cycle of fermented products packaging.

## 3. Current packaging trends for food industry

The fermented food products packaging industry has witnessed remarkable growth in recent years, driven by the increasing popularity of nutraceuticals like yogurt, kefir, and other non-dairy probiotic-rich products [3]–[5]. Consumers' evolving preferences for convenience and portability have led to the rise of single-serving and on-the-go packaging formats, such as plastic cups, pouches, and bottles with resealable caps, enabling dairy enthusiasts to enjoy their favorite products anytime and anywhere. Sustainability is a paramount concern, with brands increasingly adopting eco-friendly packaging materials like recyclable plastics, compostable options, and paper-based solutions to reduce their environmental footprint [14].

As health-conscious consumers seek dairy products with added probiotics and functional ingredients, packaging that highlights the health benefits and nutritional information of these fermented dairy products has become essential [15]. These packaging trends reflect the industry's adaptability to consumer demands and its commitment to both product quality and environmental responsibility. Alongside these trends, a comprehensive overview of sustainable packaging materials commonly employed for fermented dairy products is presented in Table 1.

**Table 1.** Key sustainable packaging materials for short-shelf life fermented food products.

Plastic type	Composition	Key properties	Common application	Recyclability	References
Polyethylene (PE)	Ethylene polymer	Lightweight, flexible, durable, and recyclable	Yogurt cups, milk, butter-milk, and kefir bottles	Widely accepted	[16]
Polyethylene terephthalate (PET)	Ethylene polymer with terephthalate	Excellent barrier to moisture and gas highly transparent, recyclable	Milk bottles, cheese packaging, and yogurt cups	Widely accepted	[17]
High-density polyethylene (HDPE)	Ethylene polymer with high density structure	Resistant to UV and chemicals, recyclable-moderate	Yogurt cups, butter and margarine packaging	Commonly accepted	[16]
Low-density polyethylene (LDPE)	Ethylene polymer with low density structure	Lightweight flexible and tough recyclable-limited	Food and Snacks Wraps, yogurt pouches	Limited acceptance	[16]
Polyvinyl chloride (PVC)	Vinyl chloride polymer	Excellent barrier to moisture and gas good chemical resistance, recyclable-limited	Clear dairy product containers, milk bottles	Limited acceptance	[18]
Polypropylene (PP)	Propylene polymer	Heat resistant, excellent barrier to moisture and resistant to grease and chemicals	Yogurt cups, Margarine packaging	Limited acceptance	[16]

The contemporary food landscape is marked by the fast-paced lives of consumers who prioritize convenience and portability. In response to this trend, the fermented dairy products packaging industry has witnessed a substantial shift towards packaging formats that cater to these preferences. Single-serving and on-the-go packaging solutions have become exceedingly popular, reshaping how fermented products are presented and consumed [19].

Single-serving packaging options, such as small plastic cups, have proliferated on store-shelves. These individual portions offer consumers a hassle-free and portion-controlled experience. Whether it is a cup of probiotic-rich yogurt for breakfast or a mid-day snack of kefir, the single-serving containers provide the perfect balance between convenience and food portion management [20]–[22].

Packaging innovation has brought forth resealable caps on bottles and pouches, enhancing the versatility of packaging for fermented food or beverage products [23]. These caps not only ensure the freshness of the product between servings but also enable consumers to enjoy their favorite dairy items on-the-go without the risk of spillage or waste [24].

Sustainability has emerged as a paramount concern for consumers and the dairy industry alike [25]. As the environmental consciousness deepens, brands are increasingly prioritizing eco-friendly packaging materials to reduce their carbon footprint and resonate with environmentally-conscious consumers [26].

Reviewing the issue of plastic waste generation and management in food packaging industries, one prominent approach towards sustainable packaging involves the use of recyclable plastics [27]. Several brands are adopting polyethylene terephthalate (PET) and high-density olyethylene (HDPE) plastics, among others, which can be efficiently recycled. The environmental impacts of end-of-life options of biobased and fossil-based PET and HDPE packaging have been reviewed [28]. This not only minimizes the environmental impact but also aligns with the circular economy principles, where plastics are collected, recycled, and repurposed.

Compostable packaging materials, such as biodegradable plastics or plant-based materials like polylactic acid (PLA), are gaining the attraction of researchers for the biosynthesis of lactic acid from potato-processing wastes [29]. These materials decompose naturally, reducing the burden on landfills and offering an eco-friendly alternative for consumers seeking to minimize their environmental footprint. Design of innovative and sustainable food packaging integrates techno-environmental and circular economy criteria. In this direction, polyhydroxybutyrate (PHB) a polyhydroxyalkanoate (PHA), a polymer belonging to the polyesters class that is of interest, and can be bio-derived from cellulosic wastes for application in biodegradable plastics [30].

Paper-based packaging has made a resurgence in the dairy industry, offering a biodegradable and renewable alternative to conventional plastics. Cartons and boxes made from responsibly sourced paper are being used for fermented dairy products, promoting the use of sustainable raw materials [31].

Consumers are increasingly inclined to support brands that demonstrate a commitment to sustainability through their packaging choices. Brands that prioritize environmentally responsible packaging not only appeal to eco-conscious consumers but also contribute to a positive brand image and differentiation in a competitive market [32],[33].

In essence, current packaging trends in the fermented dairy products industry reflect a careful balance between convenience and sustainability. Brands are aligning their strategies with the changing lifestyles and preferences of consumers, ensuring that their products are not only convenient and portable but also environmentally responsible. As these trends continue to evolve, packaging innovation will play a pivotal role in shaping the future of the industry, meeting consumer expectations, and addressing environmental concerns.

## 4. Emerging technologies

**Figure 2.**
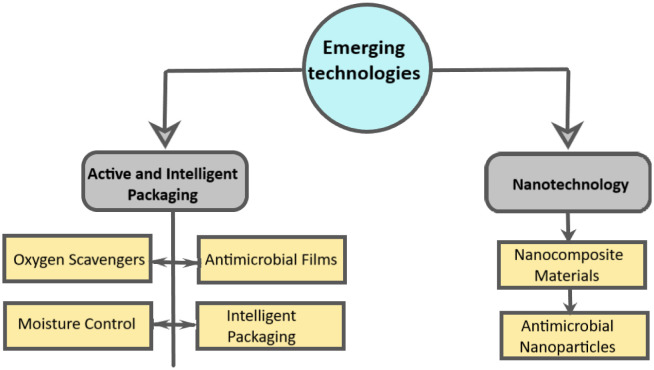
Integration of emerging packaging technologies in food production.

Emerging technologies in fermented dairy product packaging represent a dynamic frontier in the dairy industry. These innovations aim to enhance product quality, extend shelf life, and reduce environmental impact. Figure 2 illustrates the key stages involved in adopting these cutting-edge packaging solutions, providing a roadmap for dairy manufacturers seeking to integrate these advancements into their processes.

The packaging industry is on the point of transformation with the integration of active and intelligent packaging technologies for fermented food products. These innovations have the potential to significantly extend the shelf life of functional food products such as probiotic beverages prepared from fermented fruits, vegetables, and cereals [34], while enhancing their safety from microbial contaminations [35]. Oxygen is a primary culprit when it comes to deteriorating the freshness of dairy products. Active packaging solutions incorporate oxygen scavengers, which are chemical agents designed to absorb oxygen within the packaging environment. By reducing oxygen levels, these scavengers stop the oxidative processes responsible for product spoilage, ultimately preserving the product's taste, texture, and nutritional value [35],[36].

Maintaining an optimal moisture level is crucial for the quality and safety of fermented dairy products. Active packaging systems can regulate moisture content by absorbing excess moisture or releasing moisture as needed. This moisture control helps prevent product deterioration, such as moisture-induced clumping or the growth of unwanted microorganisms [37]. Antimicrobial films integrated into packaging materials offer an additional layer of protection against harmful microorganisms. For such applications, antimicrobial natural compounds have been isolated from edible plant material like aniseed and their techno-economic feasibility has been assessed for industrial-scale application [38]. These films release antimicrobial agents over time, inhibiting the growth of bacteria and pathogens that could compromise product safety. This technology not only extends shelf life but also enhances product safety and quality [39]. Beyond preserving product freshness, intelligent packaging technologies are enhancing transparency and trust among consumers. Packaging equipped with QR (quick-response) codes or RFID (Radio Frequency Identification-a wireless system comprised of two components: tags and readers) tags provides real-time information about product freshness, origin, and handling [40]. Consumers can scan these codes with their smartphones to access data such as expiration dates, nutritional information, and even the product's journey from the dairy farm to the store shelf [41]. This transparency fosters consumer confidence in the product's quality and authenticity.

## 5. Nanotechnology

Nanotechnology represents a groundbreaking frontier in packaging materials, nanocellulose-based food packaging is offering novel opportunities to enhance the packaging's functional properties and their overall performance [42]. In recent studies, the cellulose nanocomposite film has shown great potential to ease the environmental pollution and human health issues caused by non-degradable petroleum-based plastic packaging. A sustainable and hydrophobic high-performance all-green pineapple peel cellulose nanocomposite film for food packaging has been designed by incorporating natural carnauba wax and cellulose nanofibers into a cellulose matrix derived from pineapple peel [43].

Nanocomposite materials consist of nanoparticles dispersed within a matrix material. In the context of fermented product packaging, these materials can significantly improve barrier properties. By incorporating nanoparticles, such as nano clays or nanosilver, packaging materials become more effective at blocking oxygen and moisture infiltration [44]. This, in turn, reduces the need for excessive packaging layers, minimizing material usage and environmental impact [45].

Nanoparticles with antimicrobial properties can be employed to develop coatings or additives for packaging materials. Nanocrystals of carboxymethyl cellulose/cellulose immobilized silver nanoparticles have been reported as an effective coating to improve the barrier and antibacterial properties of paper for food packaging applications [46]. A metallic nanoparticle integrated ternary polymer blend of PVA/starch/glycerol has been studied as a promising antimicrobial food packaging material. These coatings release antimicrobial agents, like silver, copper, and zinc nanoparticles, which actively combat the growth of harmful microorganisms on the packaging surface [47]. As a result, the risk of contamination is substantially reduced, strengthening product safety and quality assurance.

The emerging technologies in the fermented dairy products packaging industry are poised to revolutionize the sector. Active packaging solutions combat issues related to oxygen and moisture, preserving product freshness and safety. Intelligent packaging enhances consumer trust by providing real-time information about product status. Meanwhile, nanotechnology-driven innovations improve packaging materials' barrier properties and introduce antimicrobial capabilities, promising enhanced product quality and sustainability. As these technologies mature and become more widespread, they are likely to play a pivotal role in shaping the future of packaging for fermented dairy products.

## 6. Sustainability initiatives-innovations in bioplastics

As sustainability takes the center stage in the dairy packaging industry, therefore, dairy product manufacturers are making substantial strides by embracing recyclable and biodegradable packaging materials. These eco-conscious alternatives are pivotal in minimizing the industry's carbon footprint and reducing the environmental impact associated with packaging made of recyclable and biodegradable materials [48]. Food product manufacturers are increasingly turning to bioplastics as a sustainable alternative to traditional petroleum-based plastics. Among the prominent bioplastics are PLA and PHA, and agricultural residual materials are suitable starting substrates to produce these biobased materials [49]. These biodegradable and compostable materials are derived from renewable resources, such as corn starch or sugarcane, rather than fossil fuels [50]. They offer similar functionality to conventional plastics while possessing the distinct advantage of breaking down naturally in the environment. PLA, for instance, can be industrially composted, reducing the burden on landfills and lowering the carbon footprint associated with packaging disposal [29].

Efforts to minimize packaging waste represent a significant commitment from dairy product manufacturers toward sustainability. Several strategies are being employed to address this critical issue of the need for reduced packaging waste. Manufacturers are investing in research and development to create packaging materials that are inherently lighter in weight while retaining their structural integrity for safer transportation of products [51]. This approach reduces the overall amount of material required for packaging production, resulting in lower material consumption and lesser transportation costs [27]. Lightweight not only conserves resources but also reduces greenhouse gas emissions associated with the consumption of fuel required for long-distance transportation of products [52].

Packaging design also plays a pivotal role in the reduction of packaging-waste generated after the consumption of food products. Manufacturers are designing smaller portions, especially for single-serving products, to align with consumer preferences for portion control and convenience of purchases. Smaller portions require less packaging material while contributing to reduced waste generation [53]. Optimizing packaging shapes and dimensions is another strategy employed to minimize material usage. Irregular or inefficiently shaped packaging can lead to unnecessary wastag of material. Streamlining shapes of packaging ensures the efficient use of materials while maintaining product integrity and increasing shelf-display visual appeal to buyers [54],[55].

Food product manufacturers can collaborate with recycling facilities and waste management organizations to promote the recycling of packaging materials [56]. These collaboration initiatives with recycling facilities often involve creating recycling-friendly packaging designs and increasing the accessibility of recycling options for consumers [57]. Raising consumer awareness about responsible packaging disposal is a key component of waste reduction efforts. Manufacturers and industry organizations often engage in educational campaigns to encourage consumers to reduce, recycle or compost packaging materials correctly [58].

The dairy packaging industry is making substantial strides toward sustainability through the adoption of recyclable and biodegradable materials, as well as through initiatives to reduce packaging waste. These efforts not only align with consumer expectations for environmentally responsible practices but also contribute to the industry's broader commitment to reducing its environmental footprint. As these sustainable practices continue to evolve and gain prominence, they are likely to play an increasingly pivotal role in shaping the future of dairy product packaging.

## 7. Consumers' preferences

Consumer preferences are pivotal drivers in shaping the food product packaging industry. As consumers become more health-conscious and selective in food choices, their preferences have evolved to prioritize specific product attributes. Understanding these preferences is essential for packaging designers and manufacturers aiming to meet market demand effectively.

One of the most prominent shifts in consumers' preferences within the dairy industry pertains to their health and wellness. Consumers are increasingly interested in buying dairy products, which are enriched with probiotics and functional ingredients known for their health benefits [59],[60]. They are aware with the fact that gut microbiota can be influenced by the intake of probiotics and functional foods to sustain general wellness and alleviate gut ailments like inflammation and colon cancer [61]. This growing demand has led to a surge in sale of such food products such as probiotic-rich yogurt, kefir, and fermented milk [62]. Packaging plays a pivotal role in catering to health-conscious consumers. Labels and packaging designs that prominently feature health benefits, nutritional information, and claims regarding probiotics and functional ingredients can significantly influence their purchasing decisions. Consumers often look for labels that attempt to sell attributes such as “live and active cultures”, “high in probiotics”, or “supports digestive health”, etc. A novel type of edible biomaterial composed of monomethoxyl polyethylene glycol-modified O-carboxymethyl chitosan has been studied for fresh-keeping food packaging materials [63]. Dual-crosslinked starch–poly (ester urethane)–oligochitosan films with high starch content have been suggested for their use in biodegradable food packaging [64],[65].

Health-conscious consumers may also appreciate packaging solutions that offer the convenience of purchases for controlling diet-portion sizes. Single-serving packaging options, which are both convenient for on-the-go consumption and provide precise portion sizes, align with these consumers' preferences [19],[66]. Consumers increasingly value the transparency and authenticity of the food products they choose and pay money for, mainly fermented probiotic dairy items. They prefer to seek a clear and accurate information about the sourcing of ingredients, their production methods, and overall the quality of the product purchased [67].

Packaging that provides informative labelling can foster trust and loyalty among consumers [68]. Brands that disclose details about the source of milk, production processes (e.g., traditional fermentation methods), and the absence of artificial additives or preservatives can resonate with consumers who prioritize authenticity [69]. Consumers are interested in knowing where the dairy products they consume come from. Packaging of processed food and beverage products can convey information about the sourcing of milk, including details about the farms or regions where it is produced. Such transparency can create a deeper connection between consumers and the products they choose [70]. Authenticity is not limited to product content alone but extends to a brand's commitment to environmental responsibility. Packaging materials and practices that align with sustainability and eco-friendliness can enhance a brand's authenticity by demonstrating a commitment to ethical and responsible production [71].

Understanding and responding to consumer preferences for health, wellness, transparency, and authenticity are paramount for success in the food product packaging industry. Packaging designs and messaging that align with these preferences can influence consumers' purchasing decisions, build brand loyalty, and position products favorably in a competitive market. As consumers continue to prioritize these factors, packaging strategies that cater to their evolving preferences will remain instrumental in shaping the future of the industry.

## 8. Navigating regulations: Ensuring compliance and safety

In the realm of fermented dairy product packaging, ensuring compliance with stringent regulations is of paramount importance. Regulatory bodies like the Food and Drug Administration (FDA) in the United States and the European Food Safety Authority (EFSA) in Europe have established comprehensive guidelines and standards to safeguard the quality and safety of dairy products [72]. Comprehending and adhering to these regulations is imperative for packaging designers, manufacturers, and the industry at large to avoid costly repercussions such as recalls and damage to brand reputation [73]. Regulatory agencies have defined strict guidelines for the labeling of fermented dairy products. Packaging must accurately reflect product identity, ingredients, nutritional information, and allergen declarations. Claims related to health benefits, probiotics, and functional ingredients must be substantiated with scientific evidence [74].

Detailed nutritional information, including calories, fats, carbohydrates, proteins, vitamins, and minerals, must be prominently displayed on the packaging [75]. Nutrient content claims and health claims must adhere to specific criteria, ensuring that consumers receive accurate and reliable information about the product's nutritional profile [76]. Claims related to health benefits are closely monitored and regulated [77]. Packaging that makes health-related claims must meet specific criteria and have scientific evidence to support such claims. Misleading health claims can lead to regulatory scrutiny and potential legal consequences [78]. Regulatory bodies establish stringent quality and safety standards for fermented dairy products [79]. This includes parameters for microbial safety, product composition, and limits for contaminants and pathogens. Packaging materials must meet established safety standards to prevent contamination or alteration of the product [80],[81].

Navigating the compliance challenges and regulatory requirements can pose significant challenges to packaging designers and manufacturers. Regulatory requirements can be complex and subject to change. Keeping up with evolving regulations and ensuring that packaging remains compliant can be a resource-intensive endeavour [82],[83]. International regulations can vary between countries and regions, complicating packaging design for products with global distribution. Packaging must meet the specific requirements of each market it enters [84]. Non-compliance with regulations can result in product recalls, fines, legal actions, and damage to brand reputation. Packaging designers must, therefore, take a meticulous and proactive approach to ensure compliance [85],[86].

Packaging designers must possess in-depth knowledge of relevant regulations and standards to navigate this complex landscape effectively. Collaboration with regulatory experts and legal counsel is often necessary to ensure that packaging designs align with all applicable laws and guidelines [87]. Compliance with regulatory requirements is a non-negotiable aspect of packaging design and production within the fermented dairy product industry. Packaging designers, manufacturers, and stakeholders must maintain a rigorous commitment to staying informed about and adhering to these regulations to safeguard product quality, safety, and brand integrity. Failure to meet these standards can result in severe consequences, making regulatory compliance a top priority in the industry [88].

## 9. Limitations of current packaging: Addressing the drawbacks

While the fermented dairy packaging industry has made significant advancements, several limitations persist in current packaging solutions. Recognizing and addressing these drawbacks is essential for the industry's continued growth and improvement [89].

Certain packaging materials used for fermented dairy products may have limited recyclability. This limitation contributes to environmental concerns as recycling options become restricted. Addressing this issue involves exploring more recyclable packaging alternatives and educating consumers on responsible disposal [89],[90].

Maintaining consistent temperature control during distribution is a critical aspect of ensuring product freshness and safety. Temperature fluctuations can affect the quality and shelf life of fermented dairy products. Innovative packaging solutions and distribution processes are needed to address these challenges and uphold product integrity [91],[92]. Consumer concerns regarding chemical migration from packaging materials into dairy products persist. Addressing this issue requires thorough testing and the development of packaging materials that are free from harmful chemicals, ensuring consumer safety and peace of mind [93],[94]. Solving these limitations necessitates ongoing research and innovation within the industry to develop packaging solutions that are more environmentally friendly, temperature-stable, and chemically inert.

## 10. Research gaps in packaging: Opportunities for advancement and innovation

Identifying and addressing research gaps is crucial for advancing the field of fermented dairy packaging and fostering innovation. Some key research areas that present opportunities for advancement include understanding the long-term effects of novel packaging materials on product quality and safety is essential. Research should focus on how these materials interact with dairy products over extended periods, ensuring that they do not compromise product integrity or pose any health risks [95].

Optimizing packaging design to align with circular economy principles is an emerging area of research. Developing packaging solutions that are recyclable, reusable, or compostable can contribute to a more sustainable packaging ecosystem, reducing environmental impact [96],[97]. Cost-effective solutions are needed for small-scale producers, for example, small-scale dairy producers often face unique challenges in packaging their products efficiently and cost-effectively. Research should explore innovative, cost-efficient packaging solutions tailored to the needs of small-scale producers, supporting their sustainability and competitiveness [98]. Collaborative research efforts involving academia, industry, and regulatory bodies can help fill knowledge gaps and drive innovation in fermented dairy packaging. These partnerships facilitate the exchange of expertise and resources, accelerating progress in the field [99]. By addressing these research gaps, the fermented dairy packaging industry can advance its capabilities, improve sustainability, and enhance product safety and quality. Collaborative efforts and a commitment to ongoing research are key to driving innovation and ensuring the industry's continued growth and success.

## 11. Potential developments for sustainable food packaging

The future of the fermented dairy product packaging industry is characterized by a landscape brimming with promise and potential. Anticipated developments and prospects herald a new era of innovation, sustainability, and enhanced consumer satisfaction. Here are key insights into what the future holds for this dynamic industry. Innovations are sought in active and intelligent packaging, and for that purpose, the integration of active and intelligent packaging technologies will be at the forefront of future developments. These technologies will not only extend the shelf life of fermented dairy products but also enhance their safety and appeal. Smart packaging with embedded sensors, QR codes, and RFID. Tags will provide consumers with real-time information about product freshness, origin, and handling, fostering transparency and trust [37],[100].

Sustainability will remain a paramount concern, driving the adoption of eco-friendly sustainable packaging materials. Innovations in bioplastics, such as PLA and PHA, will continue to gain momentum, providing viable alternatives to traditional plastics. Additionally, packaging materials derived from renewable resources and circular economy principles will become more prevalent, reducing the environmental footprint of dairy packaging [101]. Convenience-driven packaging designs will cater to consumers' insatiable appetite for convenience and on-the-go consumption. Single-serving and portion-controlled packaging solutions will evolve to meet the demands of modern lifestyles. These designs will not only enhance convenience but also reduce food waste, aligning with sustainability objectives [102]. Packaging will play an integral role in highlighting the health benefits of fermented dairy products. Labels and designs will emphasize probiotics, functional ingredients, and nutritional information to attract health-conscious consumers [103]. As consumers prioritize their well-being, packaging will serve as a conduit for conveying the health attributes of dairy products [104],[105].

Ensuring the maintenance of product safety will remain a paramount concern. Packaging will continue to incorporate technologies and materials that protect against contamination, temperature fluctuations, and chemical migration. These measures will uphold product quality and safety, meeting the stringent requirements of regulatory bodies and discerning consumers [106]. Meeting consumer expectations for healthier, sustainable, and convenient options will be a driving force in shaping the industry's future. Brands that align with these expectations and communicate their commitment to environmental responsibility through packaging will thrive in the evolving market landscape [107].

The fermented dairy product packaging industry stands on the verge of a transformative era. Innovations in active and intelligent packaging, sustainable materials, and convenience-driven designs will redefine the industry's standards. These developments, coupled with a steadfast commitment to maintaining product safety and meeting consumer expectations, pave the way for a promising future where dairy products are not only delicious and nutritious but also packaged with sustainability, convenience, and transparency in mind. The journey ahead is one of continued growth and evolution, with exciting possibilities for both consumers and industry stakeholders alike.

## 12. Key findings and insights

The fermented dairy products packaging industry is in the midst of a profound transformation driven by evolving consumer preferences. Consumers are increasingly seeking health-conscious, sustainable, and convenient options, igniting innovation in packaging materials and technologies. Brands that align with these preferences are poised for success in this dynamic market. Sustainability has surged to the forefront, prompting the adoption of recyclable and biodegradable materials and concerted efforts to minimize packaging waste. Eco-friendly packaging solutions resonate with consumers and contribute to a more environmentally responsible industry.

Advancements in technology are extending product shelf life and enhancing safety through active and intelligent packaging solutions. These innovations not only ensure product freshness but also foster transparency and trust among consumers via real-time information access. Nanotechnology holds promise, particularly in enhancing packaging materials' barrier properties and introducing antimicrobial coatings, bolstering both product quality and safety. Consumer demand for transparency is reshaping packaging designs, with brands providing clear and accurate information about sourcing, production methods, and ingredient quality cultivating trust and loyalty. However, navigating stringent regulations and ensuring compliance is non-negotiable, requiring packaging designers and manufacturers to possess a deep understanding of regulatory requirements to avoid costly consequences and protect brand reputation.

Although progress has been made, certain limitations persist, such as recyclability challenges, temperature control during distribution, and concerns about chemical migration, underscoring the importance of addressing these drawbacks for the industry's continued growth and sustainability. Identifying research gaps and investing in collaborative research efforts are essential for pushing the boundaries of packaging innovation. These efforts encompass understanding the long-term effects of novel materials, optimizing packaging for circular economy principles, and providing cost-effective solutions for small-scale producers.

The future of fermented dairy product packaging holds immense promise, featuring innovations in active and intelligent packaging, sustainable materials, and convenience-driven designs. Meeting consumer expectations for healthier, sustainable, and convenient options while upholding product safety will be central to future developments. In conclusion, the fermented dairy packaging industry is on an exciting trajectory of growth and evolution, benefiting both consumers seeking quality and convenience and industry stakeholders striving for sustainability and growth.

## 13. Conclusion

The fermented dairy products packaging industry is undergoing a significant transformation, shaped by dynamic shifts in consumer preferences and growing sustainability concerns. Packaging trends have evolved to prioritize convenience, sustainability, and transparency, reflecting consumers' demand for healthier and eco-friendly options. Emerging technologies are enhancing product safety and freshness, creating opportunities for innovation within the sector.

As the industry continues to expand, stakeholders must remain adaptable and innovative, constantly evolving to meet the changing demands of consumers while upholding environmental responsibility. By embracing potential developments such as active and intelligent packaging, sustainable materials, and convenient designs, the fermented dairy products packaging industry is well-positioned for a promising future. These future promises not only enhanced product quality and safety but also a more sustainable and consumer-focused approach that aligns with the values and expectations of a discerning market. As the journey unfolds, the industry's commitment to evolution and excellence will ensure its continued growth and relevance in the global marketplace.

## Use of AI tools declaration

The authors declare they have not used artificial intelligence (AI) tools in the creation of this article.
